# The complete genome sequence of a Crimean-Congo Hemorrhagic Fever virus isolated from an endemic region in Kosovo

**DOI:** 10.1186/1743-422X-5-7

**Published:** 2008-01-15

**Authors:** Darja Duh, Stuart T Nichol, Marina L Khristova, Ana Saksida, Iva Hafner-Bratkovič, Miroslav Petrovec, Iusuf Dedushaj, Salih Ahmeti, Tatjana Avšič-Županc

**Affiliations:** 1 Institute of Microbiology and Immunology, Medical Faculty, Ljubljana, Slovenia; 2 Special Pathogens Branch and Biotechnology Core Facility Branch, Centers for Disease Control and Prevention, Atlanta, Georgia, USA; 3 National Institute of Chemistry, Ljubljana, Slovenia; 4 National Institute of Public Health, Pristina, Kosovo; 5 Clinic of Infectious diseases, Pristina, Kosovo

## Abstract

The Balkan region and Kosovo in particular, is a well-known Crimean-Congo hemorrhagic fever (CCHF) endemic region, with frequent epidemic outbreaks and sporadic cases occurring with a hospitalized case fatality of approximately 30%. Recent analysis of complete genome sequences of diverse CCHF virus strains showed that the genome plasticity of the virus is surprisingly high for an arthropod-borne virus. High levels of nucleotide and amino acid differences, frequent RNA segment reassortment and even RNA recombination have been recently described. This diversity illustrates the need to determine the complete genome sequence of CCHF virus representatives of all geographically distinct endemic areas, particularly in light of the high pathogenicity of the virus and its listing as a potential bioterrorism threat. Here we describe the first complete CCHF virus genome sequence of a virus (strain Kosova Hoti) isolated from a hemorrhagic fever case in the Balkans. This virus strain was isolated from a fatal CCHF case, and passaged only twice on Vero E6 cells prior to sequence analysis. The virus total genome was found to be 19.2 kb in length, consisting of a 1672 nucleotide (nt) S segment, a 5364 nt M segment and a 12150 nt L segment. Phylogenetic analysis of CCHF virus complete genomes placed the Kosova Hoti strain in the Europe/Turkey group, with highest similarity seen with Russian isolates. The virus M segments are the most diverse with up to 31 and 27% differences seen at the nt and amino acid levels, and even 1.9% amino acid difference found between the Kosova Hoti and another strain from Kosovo (9553-01). This suggests that distinct virus strains can coexist in highly endemic areas.

## Findings

Bioinformatics analysis of complete microbial genomes has led to advances in the development of novel diagnostic techniques, in the research of microbial pathogenesis, and in the control and prevention of infectious diseases. Until the year 2006, only 2 complete genomes of Crimean-Congo hemorrhagic fever virus (CCHFV) had been sequenced [1]. CCHFV, is a tick-borne virus with tripartite RNA genome (S, M and L segment), and is the causative agent of a lethal zoonosis named Crimean-Congo hemorrhagic fever (CCHF). The virus is distributed over much of Asia, extending from China to the Middle East and Southern Russia and to the focal endemic areas in Africa and southern Europe, including Kosovo and Turkey [2]. Yearly epidemics, as well as sporadic cases of CCHF are seen in some of these areas, often with high case fatality (approx. 30%) [3]. CCHFV can be transmitted to humans by bites of *Ixodid *ticks and by the contact with blood or tissue from viremic livestock and human patients [2]. Development of diagnostic approaches and potential vaccines is dependent on knowledge of the broad geographic distribution of diverse virus variants and on understanding of the extent of virus genetic reassortment and recombination [3,4]. The analysis of the 16 existing complete CCHFV genomes up to date indicated considerable evolution and high diversity of CCHFV [1,5]. Presumably this reflects the typical high polymerase error rates seen with negative stranded RNA viruses. In addition, previous reports have found evidence of RNA segment reassortment events between CCHFV M segments, and the recombination in CCHFV S segments [1,3,4]. The genetic diversity of CCHFV, its virulence, and its potential as a bioterrorism agent, make it important to obtain the complete genome of CCHFV from all geographically distinct endemic areas.

The Balkan peninsula, and Kosovo in particular, is a well-known endemic region for CCHF, and epidemic outbreaks and sporadic cases have been frequently been recorded [6-8]. Five nucleotide sequences of CCHFV from Kosovo have been published [9-12]. Three of them are partial sequences of S segment, the remaining 2 represent complete sequences of S and M segment of different CCHFV strains, Kosova Hoti and Kosovo 9553-01, respectively. We describe the first complete CCHFV genome sequence of a virus (strain Kosova Hoti) isolated from a hemorrhagic fever case in the Balkans.

The CCHFV Kosova Hoti strain was isolated from a blood of a female fatal case during the epidemic in Kosovo in 2001 [6]. The blood was taken on the 5^th ^day after onset of symptoms. Results of the laboratory analysis showed the presence of IgM antibodies (titer 1:400) and the presence of viral RNA in the concentration of 1.08 × 10^10 ^copies per mL of serum. Virus was grown on Vero E6 cells in BSL-3 laboratory. Viral RNA was extracted with the Trizol reagent from the second passage of the CCHFV in Vero E6 cells, and used for the direct sequencing of the complete genome of the virus. Amplicons of S, M and L full length segments were obtained by following the protocols described previously [1,9,13]. Briefly, a total of 16 S, 40 M and 84 L sequencing primers were used to generate the complete sequence of the S, M and L segments and these are deposited in the GenBank under the accession numbers DQ133507, EU037902 and EU044832, respectively. Sequence alignment of CCHFV Kosova Hoti strain complete genome with preexisting CCHFV genomes was performed using the CLUSTAL W algorithm of MegAlign module (Lasergene 1999, DNASTAR, USA). Phylogenetic relationships of different CCHFV strains were established with a software package TREECON [14]. The phylogenetic tree was constructed by the neighbor-joining method. The topology of the tree was obtained with the Kimura 80 model and support for the tree nodes was calculated with 500 bootstrap replicates. SignalIP was used to predict the signal sequence cleavage site and TMHMM 2.0 was used to predict transmembrane helices of M segment [15,16]. The amino acid (aa) sequence of L segment was subjected to the PSI-BLAST and PredictProtein server for search of conserved aa motifs [17,18].

The genome size of CCHFV, strain Kosova Hoti, was found to be approximately 19.2 kb in length, consisting of a 1672 nucleotide (nt) S segment, a 5364 nt M segment and a 12150 nt L segment. The open reading frame (ORF) of S segment is 1449 nt in length, encoding a 482 aa (nt position 56 – 1504) nucleocapsid protein. The ORF lengths of M and L segments are 5067 nt/1688 aa (nt position 78–5144) and 11838 nt/3945 aa (nt position 78–11915), respectively.

**Figure 1:**
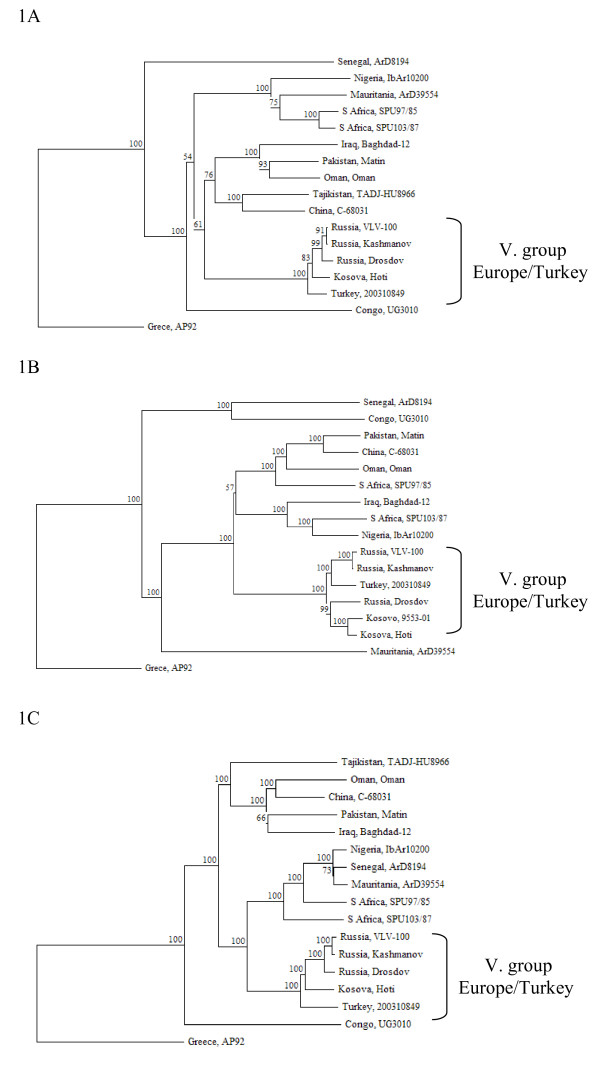
The phylogenetic analysis of the complete genome of CCHFV Kosova Hoti strain. A. Phylogenetic tree based on the alignment of S segment. B. Phylogenetic tree based on the alignment of M segment. C. Phylogenetic tree based on the alignment of L segment. CCHFV strains are presented by the country of origin and the name of the strain. Accession numbers of CCHFV used for the alignment are: Greece AP92 [S segment: GenBank:DQ211638, M segment: DQ211625, L segment: 211612], Congo UG3010 [DQ211650, DQ211637, DQ211624], Senegal ArD8194 [DQ211639, DQ211626, DQ211613], Nigeria IbAr10200 [U88410, AF467768, AY389508], Mauritania ArD39554 [DQ211641, DQ211628, DQ211615], S Africa SPU97/85 [DQ211646, DQ211633, DQ211620], S Africa SPU103/85 [DQ211647, DQ211634, DQ211621], Iraq Baghdad-12 [AJ538196, AJ538197, AY947890], Pakistan Matin [AF527810, AF467769, AY422208], Oman Oman [DQ211645, DQ211632, DQ211619], Tajikistan TADJ-HU8966 [AY049083, AY179962, AY720893], China C-68031 [DQ211642, DQ211629, DQ211616], Russia Drosdov [DQ211643, DQ211630, DQ211617], Russia Kashmanov [DQ211644, DQ211631, DQ211618], Russia VLV-100 [DQ206447, DQ206448, AY995166], Turkey 200310849 [DQ211649, DQ211636, DQ211623], Kosovo 9553-01 [M segment: AY675511].

Three phylogenetic trees were constructed based on the ORF sequences of S, M and L segments of CCHFV (Fig. 1). The general topologies of the trees were consistent with those described previously [1,13]. Seven distinct groups were formed representing the approximate geographic distribution of CCHFV. Based on the analysis of S, M and L segment of Kosova Hoti CCHFV, this strain clustered in group V., which represents the Europe/Turkey geographic lineage [1]. The position of Kosova Hoti strain within group V. was similar in the S and L segment tree (Fig. 1, panels A and C), where it formed a separate lineage ancestral to the three Russian isolates and CCHFV strain 200310849 from Turkey was the most ancestral member of V. group. The group V. topology based on the M segment, was a little different (Fig. 1, panel B). Two Russian strains (VLV-100 and Kashmanov) clustered together with the Turkish CCHFV whereas the Russian Drosdov strain clustered together with both CCHFV strains from Kosovo.

**Figure 2:**
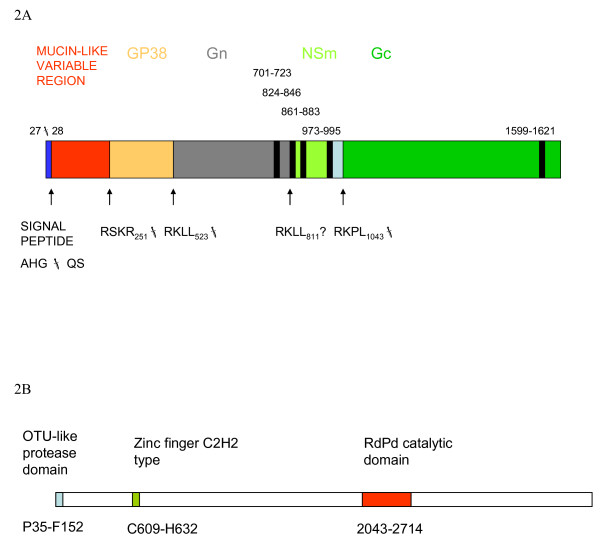
The protein analysis of the complete genome of CCHFV Kosova Hoti strain. A. Scheme of the M polyprotein of Kosova Hoti. B. Scheme of the L protein of Kosova Hoti strain.

The sequence differences between the CCHFV strains in the group V. are shown in Tables 1, 2, 3. Significant difference was noted between the nt (ORF) and aa sequences of S and L segments, in comparison to the M segment. The majority of nt changes in the S and L segments were synonymous (not amino acid changing) (Tables 1, 3), whereas over 80% of M segment nt changes were non-synonymous (amino acid changing) (Table 2). As seen in earlier studies [12,19,20], considerable glycoprotein amino acid variation was observed, particularly in the mucin-like variable region (Fig. 2), and presumably reflects the biological function of the glycoproteins encoded by the M segment. It is somewhat surprising that the glycoproteins of Kosova Hoti and another strain from Kosovo, 9553-01, differed by 1.9% in complete aa sequence, and up to 4.5% in the mucin-like domain (Table 2). This suggests different genetic strains of CCHFV co-exist in this highly endemic region.

**Table 1:** The difference between complete S segment of CCHFV strain Kosova Hoti and other strains in the V. group (Europe/Turkey) calculated by the MegAlign module.

S segment, difference (%)	Kosova Hoti			
CCHFV strain	nt sequence (complete)	nt sequence (ORF)	aa sequence	non-synonymous mutations (%)

Kosovo, 9553-01	NA	NA	NA	NA
Turkey, 200310849	2.1	2.1	0.0	0
Russia, Drosdov	2.6	2.6	0.6	23
Russia, Kashmanov	2.1	2.1	0.4	19
Russia, VLV-100	2.0	2.0	0.2	10

CCHFV strains are presented by the country of origin and the name of the strain. Accession numbers are shown in the legend of Figure 1A. nt – nucleotide; aa – amino acid, ORF – open reading frame, NA – S sequence of Kosovo 9553-01 strain not available.

**Table 2:** The difference between complete M segment of CCHFV strain Kosova Hoti and other strains in the V. group (Europe/Turkey) calculated by the MegAlign module. Table includes a separate column for the Mucin-like variable region present in M segment.

M segment, difference (%)	Kosova Hoti				
CCHFV strain	nt sequence (complete)	nt sequence (ORF)	aa sequence	non- synonymous mutations (%)	Mucin-like VR, aa 28–251 (% difference)

Kosovo, 9553-01	2.3	2.2	1.9	86	4.5
Turkey, 200310849	5.7	5.6	4.8	85	20.5
Russia, Drosdov	5.2	5.1	4.3	84	15.2
Russia, Kashmanov	5.4	5.2	4.5	86	19.6
Russia, VLV-100	5.7	5.4	4.5	83	20.1

CCHFV strains are presented by the country of origin and the name of the strain. Accession numbers are shown in the legend of Figure 1B. nt – nucleotide; aa – amino acid, ORF – open reading frame.

**Table 3:** The difference between complete L segment of CCHFV strain Kosova Hoti and other strains in the V. group (Europe/Turkey) calculated by the MegAlign module.

L segment, difference (%)	Kosova Hoti			
CCHFV strain	nt sequence (complete)	nt sequence (ORF)	aa sequence	non-synonymous mutations (%)

Kosovo, 9553-01	NA	NA	NA	NA
Turkey, 200310849	4.4	4.2	1.2	28
Russia, Drosdov	3.6	3.4	1.1	32
Russia, Kashmanov	3.5	3.4	0.9	26
Russia, VLV-100	3.7	3.5	1.0	28

CCHFV strains are presented by the country of origin and the name of the strain. Accession numbers are shown in the legend of Figure 1C. nt – nucleotide; aa – amino acid, ORF – open reading frame, NA – L sequence of Kosovo 9553-01 strain not available.

The analysis of the Kosova Hoti strain M segment encoded polyprotein predicted the cleavage of the signal peptide to occur between aa 27 and 28 (AHG-QS). This site is identical to those described for Kosovo 9553-01 and Kashmanov but differs from other strains in group V. (Fig. 2). The mucin-like variable region of Kosova Hoti strain polyprotein stretches from aa 28 to 251 and differs by up to 20.5% from Turkish 200310849 strain (Table 2). Tetrapeptides RSKR_251_, RKLL_523 _and RKPL_1043 _were identified in Kosova Hoti and are identical among all strains in V. group. They represent the cleavage sites for GP38, Gn and Gc proteins, respectively [21,22]. The RKLL_523 _tetrapeptid of Kosova Hoti is typical for all strains in group V (Europe/Turkey) but it differs from RRLL tetrapeptid in all other CCHFV strains sequenced. However, both tetrapeptides constitute a cleavage recognition site for subtilase SKI-1 [12,22,23]. Five transmembrane helices were predicted for polyprotein of Kosova Hoti as shown on Figure 2.

Analysis of L protein encoded by the L segment of the Kosova Hoti strain revealed the conserved OTU-like protease domain from aa 35 to 152 (Fig. 2). The identified sequence G_37_**D**GN_40_**C**FYHSIAE....._151_**H**FD with the catalytic triad (indicated in **bold**) was identical among all CCHFV strains used in the L segment alignment (Fig. 1, panel C). Amino-acids 2043–2714 corresponded to the RNA-dependent RNA polymerase catalytic domain, similarly to the Nigerian IbAr10200 strain [24]. In addition, a zinc finger C2H2-type domain (aa 609–632) was found in the L protein of Kosova Hoti, but a previously identified leucine zipper could not be predicted. A leucine zipper motif (composed of three heptads) previously identified at aa 1386–1407 in the L sequence of a Nigerian strain [24,25], was not identified in the Kosova Hoti L sequence. However, the L sequence of Kosova Hoti (and other strains from group V) in this region differs from the Nigerian strain only in the substitution of the leucine for isoleucine at the position 1386.

Frequently it is observed that arthropod-borne viruses of vertebrates exhibit low genetic diversity which is thought to be due to essentially a double filter in operation, whereby evolution of these viruses is tightly constrained by the need to maintain high fitness in both vertebrate and arthropod host environments [26]. The very high genetic diversity seen in CCHFV is a strikingly exception. Presumably less constraint or greater positive selection is molding the evolutionary pattern of this virus. The complete genome of this representative CCHFV isolate (Kosova Hoti) from a highly endemic region of the Balkans is clearly divergent from strains present in other endemic regions of the world, and considerable sequence difference is even observed among virus strains found within Kosovo. These findings have importance for design of molecular diagnostic tools and vaccine development efforts, as they clearly illustrate the need to consider the high viral diversity and complexity of CCHF viral variant geographic distribution in these efforts.

## Competing interests

The author(s) declare that they have no competing interests.

## Authors' contributions

DD performed RNA extraction, qualitative and quantitative RT-PCR, analyzed the data and prepared the draft manuscript. MK and STN provided the complete M and L segment sequences and revised the draft manuscript. AS sequenced the complete S segment. IHB performed the protein analysis. MP, ID and SA collected the samples and clinical data. TAZ isolated the virus, supervised the study and revised the final draft. All authors read and approved the final manuscript.
